# To New Subscribers

**Published:** 1896-08

**Authors:** 


### TO NEW SUBSCRIBERS.

Any new subscriber ordering THE JOURNAL now will get it until January 1, 1898, for $2.00. If he pays the $2.00 January, 1897, he will get any premium offered new subscribers. If he doesn't want the premium he can pay the $2.00 after October 1, 1897.

---

THE American Microscopical Society will hold its nineteenth annual meeting in the new Carnegie Library Building, Pittsburg, Pa., Tuesday, Wednesday, Thursday, and Friday, August 18, 19, 20, 21, 1896. A hearty welcome will be extended to all interested in the microscopical sciences. Applications for membership and titles of papers to be read at the meeting should be addressed, to A. Clifford Mercer, M.D., President, Syracuse, N. Y., or to Wm. C. Krauss, M.D., Secretary, 382 Virginia street, Buffalo, N. Y.

---

A DEATH from chloroform is reported in Pensacola, Fla. Dr. J. H. Pierpont was administering the anesthetic, and some operation was being done by Dr. W. E. Anderson. Only a small quantity of chloroform had been used.

---

INFORMATION concerning various Medical Colleges, their teachers and programs, will be found on pages XXI to XXIX.

